# Bovine α-Lactalbumin Hydrolysates (α-LAH) Ameliorate Adipose Insulin Resistance and Inflammation in High-Fat Diet-Fed C57BL/6J Mice

**DOI:** 10.3390/nu10020242

**Published:** 2018-02-23

**Authors:** Jing Gao, Jiajia Song, Min Du, Xueying Mao

**Affiliations:** 1 Beijing Advanced Innovation Center for Food Nutrition and Human Health, College of Food Science & Nutritional Engineering, China Agricultural University, Beijing 100083, China; gaokeke1992@126.com (J.G.); songjiajia1208@126.com (J.S.); 2 College of Food Science and Nutritional Engineering, Key Laboratory of Functional Dairy, Ministry of Education, China Agricultural University, Beijing 100083, China; 3 Department of Animal Sciences, Washington State University, Pullman, WA 99164, USA; min.du@wsu.edu

**Keywords:** insulin resistance, inflammation, bovine α-lactalbumin hydrolysates, high-fat diet, adipose tissue

## Abstract

Obesity-induced adipose inflammation has been demonstrated to be a key cause of insulin resistance. Peptides derived from bovine α-lactalbumin have been shown to inhibit the activities of dipeptidyl peptidase IV (DPP-IV) and angiotensin converting enzyme (ACE), scavenge 2,2′-azinobis [3-ethylbenzothiazoline-6-sulfonate] (ABTS^+^) radical and stimulate glucagon-like peptide-2 secretion. In the present study, the effects of bovine α-lactalbumin hydrolysates (α-LAH) on adipose insulin resistance and inflammation induced by high-fat diet (HFD) were investigated. The insulin resistance model was established by feeding C57BL/6J mice with HFD (60% kcal from fat) for eight weeks. Then, the mice were fed with HFD and bovine α-LAH of different doses (100 mg/kg b.w., 200 mg/kg b.w. and 400 mg/kg b.w.) for another 12 weeks to evaluate its protective effects against HFD-induced insulin resistance. The oral glucose tolerance test (OGTT) and intraperitoneal insulin tolerance test (ipITT) were conducted after intervention with α-LAH for 10 weeks and 11 weeks, respectively. Results showed that bovine α-LAH significantly reduced body weight, blood glucose, serum insulin, and HOMA-IR (homeostatic model assessment of insulin resistance) levels, lowered the area-under-the-curve (AUC) during OGTT and ipITT, and downregulated inflammation-related gene [tumor necrosis factor (TNF)-α, interleukin (IL)-6, monocyte chemoattractant protein (MCP)-1] expression in adipose tissues of HFD-fed C57BL/6J mice. Furthermore, bovine α-LAH also suppressed insulin receptor substrate 1 (IRS-1) serine phosphorylation (Ser307, Ser612), enhanced protein kinase B (known as Akt) phosphorylation, and inhibited the activation of inhibitor of kappaB kinase (IKK) and mitogen activated protein kinase (MAPK) signaling pathways in adipose tissues of HFD-fed C57BL/6J mice. These results suggested that bovine α-LAH could ameliorate adipose insulin resistance and inflammation through IKK and MAPK signaling pathways in HFD-fed C57BL/6J mice.

## 1. Introduction

Obesity characterized by an excessive fat accumulation may lead to a number of metabolic disorders, such as insulin resistance, type 2 diabetes, dyslipidemia, and hypertension. Among all of these, insulin resistance is a key etiological cause [1,2,3,4].

The mechanisms by which obesity cause insulin resistance are complicated and still not completely understood, but a lot of evidence supports the view that obesity-induced inflammation may be primarily responsible for insulin resistance [5]. In obesity, the insulin resistant state is always accompanied by the increase of inflammatory cytokines including tumor necrosis factor (TNF)-α, interferon (IFN-γ), interleukin (IL)-1β, interleukin (IL)-6, and monocyte chemoattractant protein (MCP)-1. Correspondingly, the suppression of inflammatory cytokines attenuates insulin resistance [6,7]. Furthermore, the mice lacking TNF-α, IL-1β or MCP-1 exhibited improved insulin sensitivity although fed with a high-fat diet (HFD) [8,9,10]. These findings suggest that the obesity-induced insulin resistance can be ameliorated through the reduction of inflammation.

Although, in obesity, the peripheral tissues including adipose tissue, liver, and skeletal are sites of inflammation, adipose tissue plays a key role in the obesity-induced inflammation through secreting pro-inflammatory cytokines [11,12]. Accumulating data demonstrate that adipose inflammation and insulin resistance are associated with inhibitor of kappaB kinase (IKK) and mitogen activated protein kinase (MAPK) signaling pathways [13,14]. They could stimulate the production of pro-inflammatory cytokines and enhance the phosphorylation of insulin receptor substrate 1 (IRS-1) on serine residues, thus causing insulin resistance [15,16]. Hence, the IKK and MAPK signaling pathways may be the underlying mechanisms related to insulin resistance and inflammation.

Bovine α-lactalbumin is the second major whey protein in bovine milk, which plays an important role in the properties and physiological function of whey proteins [17,18,19]. It has been shown to be capable of attenuating inflammation. It suppressed IL-6 release induced by ischemia/reperfusion in rats [20], and lowered TNF-α and IL-1β levels in the colon tissues of azoxymethane and dextran sodium sulfate treated mice [21]. Bovine α-lactalbumin possesses the ability of regulating glucose homeostasis. In Wistar rats, α-lactalbumin-rich whey concentrate supplementation reversed glucose intolerance and insulin resistance in the muscle and liver induced by a high-sucrose diet [22]. In Gottingen minipigs, bovine α-lactalbumin reduced serum haptoglobin and C-reactive protein level, resulting in the amelioration of Western diet-induced glucose intolerance [23]. Furthermore, peptides derived from bovine α-lactalbumin inhibited dipeptidyl peptidase 4 (DPP-4) activity, suppressed angiotensin converting enzyme (ACE) activity, scavenged 2,2′-azinobis [3-ethylbenzothiazoline-6-sulfonate] (ABTS^+^) radical and stimulated glucagon-like peptide-2 secretion [24,25,26,27,28]. However, there are few studies about the efficacy and underlying mechanisms of bovine α-lactalbumin hydrolysates against insulin resistance.

Thus, the present study was performed to investigate the effects of bovine α-lactalbumin hydrolysates (α-LAH) on HFD induced insulin resistance and inflammation in C57BL/6J mice. To determine the underlying mechanisms of bovine α-LAH, the effects of bovine α-LAH on IRS-1/PI3K/Akt, IKK and MAPK signaling pathways were also investigated.

## 2. Materials and Methods

### 2.1. Materials

Bovine α-lactalbumin was provided by Davisco Foods International Inc. (Eden Prairie, MN, USA). Alcalase was purchased from Novozymes Biologicals Inc. (Bagsvaerd, Denmark). Insulin was purchased from Sigma-Aldrich (St. Louis, MO, USA). The mouse insulin enzyme-linked immunosorbent assay (ELISA) kit was obtained from Mercodia (Uppsala, Sweden). The primary antibody against β-actin (No. ab8227) was purchased from Abcam (Cambridge, UK). The primary antibodies against p-IRS-1 (Ser307) (No. 2381), p-IRS-1 (612) (No. 2386), IRS-1 (No. 3407), p-Akt (Ser473) (No. 4060), Akt (No. 4691), p-IKKα/β (No. 2597), IKKα (No. 2682), IKKβ (No. 2370), p-p38 (Thr180/Tyr182) (No. 4511), p38 (No. 8690), phospho-extracellular signal regulated kinases (p-ERK) (Thr202/Tyr204) (No. 4370), ERK (No. 4696), phospho-c-Jun N-terminal kinases (p-JNK) (Thr183/Tyr185) (No. 4668), JNK (No. 9252), and horseradish peroxidase-conjugated anti-rabbit secondary antibody (No. 7074) were obtained from Cell Signaling Technology (Beverly, MA, USA). All other chemicals used in the study were at least of analytical grade.

### 2.2. Preparation of Bovine α-Lactalbumin Hydrolysates (α-LAH)

Bovine α-lactalbumin was dissolved in distilled water at a concentration of 5.0% (*w*/*v*). The solutions were preincubated at 85 °C for 15 min, and then hydrolyzed with alcalase at an enzyme/substrate mass radio of 1/20 at optimal conditions (pH 8.5, 55 °C) for 4 h. After heating at 85 °C for 20 min to inactivate the protease, the hydrolysates were centrifuged at 4000× *g* for 20 min at 4 °C. The supernatants were freeze-dried and stored at −20 °C for further investigation.

### 2.3. Animals and Diets

The C57BL/6J mice (5 weeks old, male) purchased from Beijing Vital River Laboratory Animal Technology Co., Ltd. (Beijing, China) were housed three per cage at controlled temperature (22 ± 1 °C) and humidity (40% ± 10%) with a 12-h light-dark cycle. All mice had free access to food and water. After fed a normal chow diet for acclimation for 1 week, one group of the mice (*n* = 10) were fed with normal control diets (13.40% of calories from fat, 60.85% of calories from carbohydrates, 25.75% of calories from protein) (Beijing KeAo Feed Co. Ltd., Beijing, China) and the others were fed with high-fat diets (60.08% of calories from fat, 20.04% of calories from carbohydrates, 20.05% of calories from protein) for 8 weeks. Then, the mice fed normal control diets were maintained for 12 weeks (ND) and the mice fed high-fat diets were randomly divided into 4 groups of ten mice each and maintained for 12 weeks: (1) HFD mice fed high-fat diets only; (2) HFD + α-LAH100 mice fed high-fat diets with α-LAH supplementation of low dose (100 mg/kg b.w., once a day); (3) HFD + α-LAH200 mice fed high-fat diets with α-LAH supplementation of medium dose (200 mg/kg b.w., once a day); and (4) HFD + α-LAH400 mice fed high-fat diets with α-LAH supplementation of high dose (400 mg/kg b.w., once a day). The mice in ND and HFD group were administered with sterile physiological saline and the mice in other groups were administered with bovine α-LAH dissolved in sterile physiological saline by orally gavage once a day. The body weight of the mice was recorded once a week, and the food intake was measured every three days. At the end of the experiment, all mice were euthanized after 12 h of fasting. Blood samples were collected, left at 4 °C for overnight, centrifuged at 1000× *g* at 4 °C for 20 min, the separated serum was collected and stored at −80 °C until biochemical analysis. Epididymal adipose tissues were promptly dissected out, rinsed off with saline, and stored at −80 °C until further analysis. All animal experiments were approved by the Animal Ethics Committee at China Agricultural University (the ethical review serial number is KY160049).

### 2.4. Fasting Blood Glucose, Fasting Serum Insulin and HOMA-IR (Homeostatic Model Assessment of Insulin Resistance)

After an overnight fast, the blood samples were collected from the tail to measure blood glucose with a glucose meter (Roche Diagnostics, Mannheim, Germany). The serum insulin concentration was determined using a mouse insulin enzyme-linked immunosorbent assay (ELISA) kit according to the manufacturer’s instruction. HOMA-IR (homeostatic model assessment of insulin resistance) was calculated according to the following formula as previously described [29]:

HOMA-IR = [fasting glucose (mM) × fasting insulin (mU/L)]/22.5.

### 2.5. Oral Glucose Tolerance Test (OGTT)

Oral glucose test was conducted after intervention with α-LAH for 10 weeks according to the methods previously described [30]. After an overnight fast, mice were administered glucose (2 g/kg b.w., oral). Blood samples were collected from the tail before (0 min) and 30, 60, 90 and 120 min after glucose administration to measure blood glucose with a glucose meter (Roche Diagnostics, Mannheim, Germany).

### 2.6. Intraperitoneal Insulin Tolerance Test (ipITT)

Intraperitoneal insulin tolerance test was conducted after intervention with α-LAH for 11 weeks using the methods previously described [31]. After a 6-h fast, mice were administered insulin (0.75 U/kg b.w., intraperitoneal injection). Blood samples were collected from the tail before (0 min) and 30, 60, 90 and 120 min after insulin injection to measure blood glucose with a glucose meter (Roche Diagnostics, Mannheim, Germany).

### 2.7. Reverse Transcription Quantitative Real-Time PCR (RT-qPCR)

The total RNA was isolated from the epididymal adipose tissues with Trizol reagent (Tiangen Biotech, Beijing, China), and conversed to cDNA by using EasyScript Plus cDNA Synthesis Kit (ABM, Richmond, BC, Canada). The reverse transcription quantitative real-time PCR (RT-qPCR) was performed with a Techne Quantica real-time PCR detection system (Techne, Stone, Staffordshire, UK). The thermal profile for the real-time PCR was 95 °C for 180 s, followed by 40 cycles of 95 °C for 5s, 60 °C for 30 s, and 72 °C for 30 s. In addition, the primers for TNF-α, IL-6 and MCP-1 were synthesized by Invitrogen (Carlsbad, CA, USA) shown in Table 1. All results were normalized with respect to the expression of glyceraldehyde-3-phosphate dehydrogenase (GAPDH) and were presented as the fold change of each sample group relative to the ND group.

### 2.8. Western Blot Analysis

Epididymal adipose tissues were homogenized in RIPA buffer (Beyotime, Haimen, Jiangsu, China) containing 1% protease inhibitor (Sigma-Aldrich, St. Louis, MO, USA) and 1% phosphatase inhibitor (Sigma-Aldrich, St. Louis, MO, USA) to extract protein. Samples were centrifuged at 12,000× *g* at 4 °C for 15 min to collect the supernatant. Protein concentration was determined using the bicinchoninic acid protein assay kit (Tiangen Biotech, Beijing, China). The proteins were separated by 10% sodium dodecyl sulfate polyacrylamide gel electrophoresis (SDS-PAGE) and transferred onto polyvinylidene fluoride (PVDF) membranes (Millipore, Bedford, MA, USA) with a wet transfer apparatus (Bio-Rad, Hercules, CA, USA). After blocking the PVDF membranes with 5% fat-free milk in Tris-buffered saline (TBS) containing 0.1% Tween 20 (TBS-T) for 2 h at room temperature, the membranes were incubated with primary antibodies overnight at 4 °C. Next, the PVDF membranes were washed three times and incubated with a horseradish peroxidase conjugated secondary antibody for 1 h at room temperature. The enhanced chemiluminescence reagent (Millipore, Bedford, MA, USA) and autoradiographic film were used to detect the protein bands. The immunoblotted band intensity was quantified by ImageJ software (version, National Institutes of Health, Bethesda, MD, USA).

### 2.9. Statistical Analysis

All data were expressed as means ± standard deviations (SD) values. Differences between groups were evaluated using one-way ANOVA followed by Duncan’s multiple-comparison test (SPSS version 20.0, IBM Inc., Chicago, IL, USA). *p* < 0.05 was considered as statistically significant.

## 3. Results

### 3.1. Bovine α-Lactalbumin Hydrolysates (α-LAH) Prevented Body Weight Gain in HFD-Fed C57BL/6J Mice

**Figure 1:**
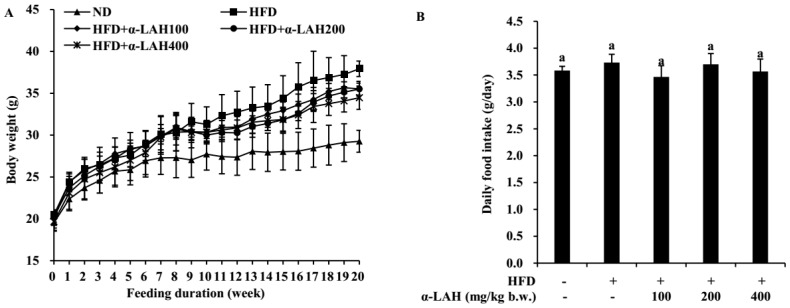
Effect of bovine α-lactalbumin hydrolysates (α-LAH) supplementation on body weight and food intake in mice fed with high-fat diet. (**A**) Changes in body weight through the experimental period; (**B**) Daily food intake. All data were presented as means ± standard deviations (SD) (*n* = 10). Significance was determined by one-way analysis of variance (ANOVA) with the Duncan’s multiple-comparison test, and mean values without common letters indicate differences at *p* < 0.05.

Compared to the mice in the ND group, high-fat diet feeding induced a marked increase in body weight from week 7 (Figure 1A). Bovine α-LAH supplementation at 100 mg/kg b.w., 200 mg/kg b.w. and 400 mg/kg b.w. significantly reversed this change, as shown by significantly lower body weight from week 16 than mice in the HFD group (Figure 1A). Meanwhile, there was no significant difference in food intake among all the experimental groups (*p* > 0.05, Figure 1B).

### 3.2. Bovine α-Lactalbumin Hydrolysates (α-LAH) Ameliorated Hyperglycemia and Hyperinsulinemia in HFD Fed C57BL/6J Mice

**Figure 2:**
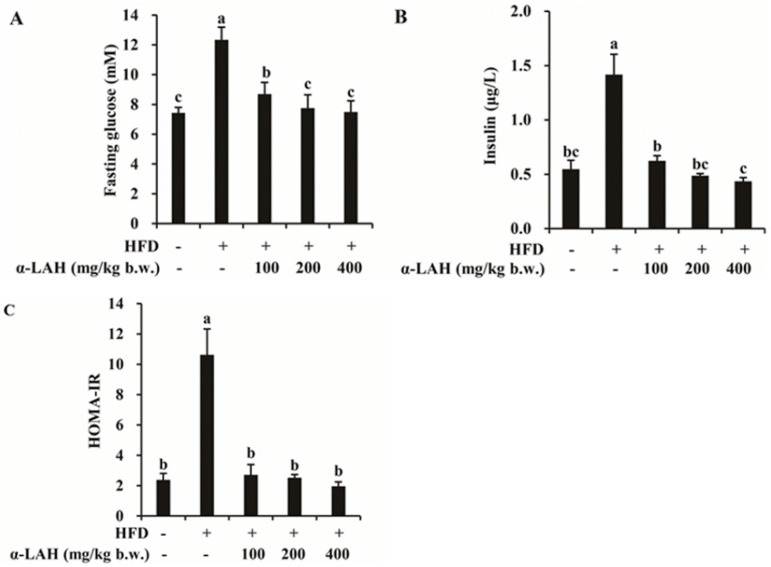
Effect of bovine α-lactalbumin hydrolysates (α-LAH) supplementation on hyperglycemia and hyperinsulinemia in mice fed with high-fat diet. (**A**) Fasting blood glucose level; (**B**) fasting serum insulin level; (**C**) Homeostatic model assessment of insulin resistance (HOMA-IR) index. All data were presented as means ± standard deviations (SD) (*n* = 10). Significance was determined by one-way analysis of variance (ANOVA) with the Duncan’s multiple-comparison test, and mean values without common letters indicate differences at *p* < 0.05.

The fasting glucose levels of the mice in the HFD group were significantly higher than that in the ND group (Figure 2A). However, the supplementation of bovine α-LAH at different doses markedly reduced the fasting glucose levels induced by HFD (Figure 2A). Similarly, HFD caused a significant increase of fasting insulin levels, and the supplementation of bovine α-LAH strongly suppressed the hyperinsulinemia (Figure 2B). In addition, bovine α-LAH supplementation suppressed the increase of HOMA-IR induced by HFD (Figure 2C). Because hyperglycemia and hyperinsulinemia are the main indicators of insulin resistance, and HOMA-IR is a direct index to measure the level of insulin resistance, these data suggested that bovine α-LAH might have protective effects on HFD induced insulin resistance.

### 3.3. Bovine α-Lactalbumin Hydrolysates (α-LAH) Improved Glucose and Insulin Tolerance in HFD-Fed C57BL/6J Mice

**Figure 3:**
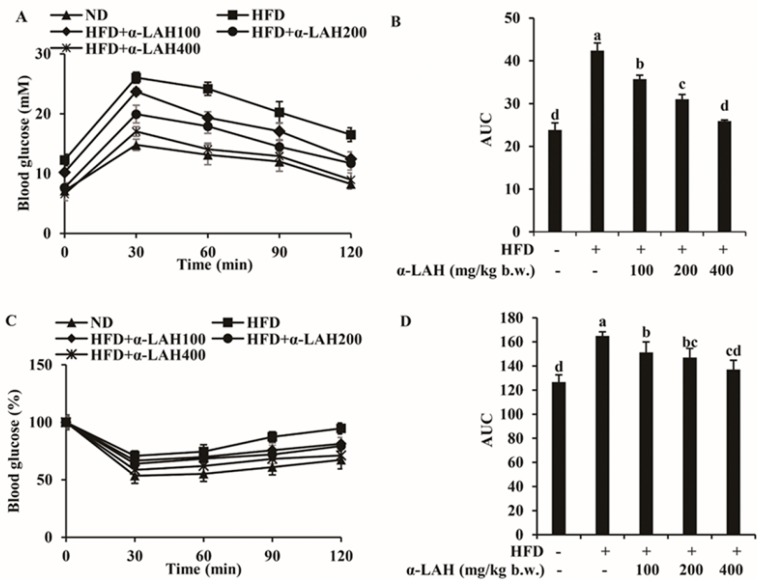
Effect of bovine α-lactalbumin hydrolysates (α-LAH) supplementation on glucose tolerance and insulin tolerance in mice fed with high-fat diet. (**A**) The results of oral glucose tolerance test (OGTT). After an overnight fast, mice were administered glucose (2 g/kg b.w., oral). Blood samples were collected from the tail before (0 min) and 30, 60, 90 and 120 min after glucose administration to measure blood glucose; (**B**) area under the curve (AUC) for the blood glucose levels during oral glucose tolerance test (OGTT); (**C**) the results of intraperitoneal insulin tolerance test (ipITT). After a 6-h fast, mice were administered insulin (0.75 U/kg b.w., intraperitoneal injection). Blood samples were collected from the tail before (0 min) and 30, 60, 90 and 120 min after insulin injection to measure blood glucose; (**D**) area under the curve (AUC) for the blood glucose levels during intraperitoneal insulin tolerance test (ipITT). All data were presented as means ± standard deviations (SD) (*n* = 10). Significance was determined by one-way analysis of variance (ANOVA) with the Duncan’s multiple-comparison test, and mean values without common letters indicate differences at *p* < 0.05.

The effects of bovine α-LAH supplementation on HFD induced glucose and insulin intolerance were investigated. After glucose administration, the blood glucose of HFD group sustained at high levels and the area-under-the-curve (AUC) during oral glucose tolerance test (OGTT) was significantly higher compared to the ND group (Figure 3A,B). The supplementation of bovine α-LAH exerted protective effect on the HFD induced glucose intolerance in a dose-dependent manner, as shown by the advanced glucose disposal and lower AUC compared to the HFD group (Figure 3A,B). Meanwhile, HFD feeding impaired insulin tolerance in mice, which was demonstrated by the attenuated action of insulin to lower glucose and the increase of AUC compared to the ND group (Figure 3C,D). When the HFD-fed mice were supplemented with bovine α-LAH, the AUC during intraperitoneal insulin tolerance test (ipITT) was markedly lower than that in the HFD group, suggesting that bovine α-LAH supplementation improved the insulin tolerance in HFD-fed mice (Figure 3C,D).

### 3.4. Bovine α-Lactalbumin Hydrolysates (α-LAH) Suppressed IRS-1 (Ser307, Ser612) Phosphorylation and Enhanced Akt (Ser473) Phosphorylation in Epididymal Adipose Tissues of HFD Fed C57BL/6J Mice

**Figure 4:**
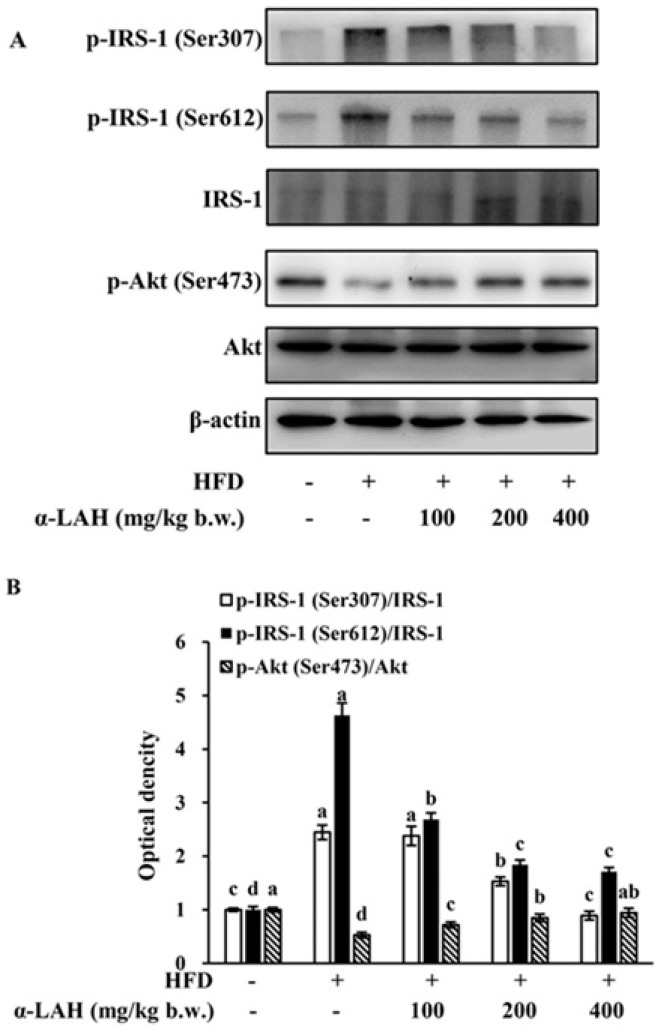
Effect of bovine α-lactalbumin hydrolysates (α-LAH) supplementation on expression of insulin signaling markers in epididymal adipose tissues of mice fed with high-fat diet. (**A**) a representative image of western blot analysis for phospho-insulin receptor substrate 1 (p-IRS-1) and p-Akt in epididymal adipose tissues; (**B**) fold change in relative densitometric levels of p-IRS-1/IRS and p-Akt/Akt relative to the ND group. All data were presented as means ± standard deviations (SD) (*n* = 6). Significance was determined by one-way analysis of variance (ANOVA) with the Duncan’s multiple-comparison test, and mean values without common letters indicate differences at *p* < 0.05.

The effects of bovine α-LAH on expression of insulin signaling markers in epididymal adipose tissues of mice fed with HFD were examined. As shown in Figure 4A,B, after fed with HFD for 20 weeks, the serine phosphorylation of IRS-1 (Ser307, Ser612) was significantly increased, but the serine phosphorylation of Akt (Ser473) was strongly decreased. When the HFD-fed mice were supplemented with bovine α-LAH, these changes were reversed in a dose-dependent manner. These results suggested that bovine α-LAH could suppress the phosphorylation of IRS-1 (Ser307, Ser612) and enhance the phosphorylation of Akt (Ser473) in adipose tissue, which means that bovine α-LAH could improve adipose insulin resistance induced by HFD.

### 3.5. Bovine α-Lactalbumin Hydrolysates (α-LAH) Downregulated Inflammation-Related Gene Expression in Epididymal Adipose Tissues of HFD-Fed C57BL/6J Mice

**Figure 5:**
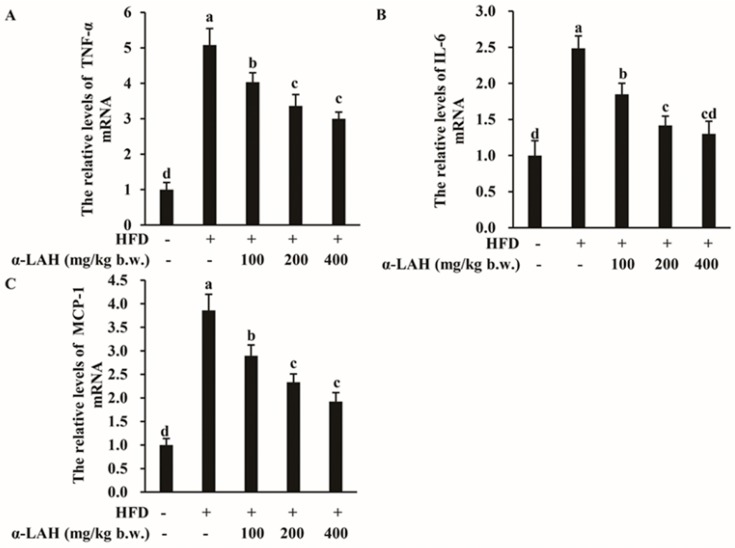
Effect of bovine α-lactalbumin hydrolysates (α-LAH) supplementation on inflammation-related gene expression in epididymal adipose tissues of mice fed with high-fat diet. (**A**) Relative levels of tumor necrosis factor (TNF)-α mRNA expression in epididymal adipose tissues; (**B**) relative levels of interleukin (IL)-6 mRNA expression in epididymal adipose tissues; (**C**) relative levels of monocyte chemoattractant protein (MCP)-1 mRNA expression in epididymal adipose tissues. All data were presented as means ± standard deviations (SD) (*n* = 6). Significance was determined by one-way analysis of variance (ANOVA) with the Duncan’s multiple-comparison test, and mean values without common letters indicate differences at *p* < 0.05.

Whether bovine α-LAH influenced the expression of inflammation-related genes including TNF-α, IL-6 and MCP-1 in epididymal adipose tissues of high-fat diet-fed C57BL/6J mice were evaluated. The mRNA expression levels of TNF-α, IL-6 and MCP-1 in epididymal adipose tissues of mice fed with HFD were significantly increased as compared to that of the ND group (Figure 5). Nevertheless, bovine α-LAH strongly downregulated the TNF-α, IL-6 and MCP-1 mRNA expression in epididymal adipose tissues of mice fed with HFD (Figure 5). These findings indicated that bovine α-LAH could ameliorate inflammation in adipose tissues induced by HFD.

### 3.6. Bovine α-Lactalbumin Hydrolysates (α-LAH) Inhibited the Activation of IKK Signaling Pathway in Epididymal Adipose Tissues of HFD Fed C57BL/6J Mice

**Figure 6:**
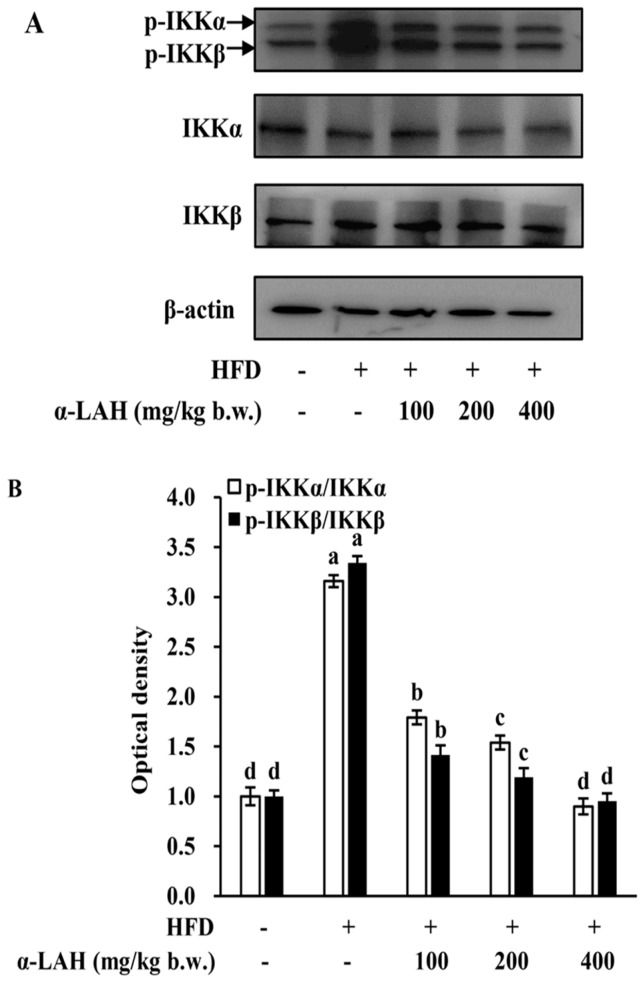
Effect of bovine α-lactalbumin hydrolysates (α-LAH) supplementation on the activation of inhibitor of kappaB kinase (IKK) signaling pathway in epididymal adipose tissues of mice fed with high-fat diet. (**A**) A representative image of western blot analysis for p-IKKα/β in epididymal adipose tissues; (**B**) fold change in relative densitometric levels of p-IKKα/IKKα and p-IKKβ/IKKβ relative to the ND group. All data were presented as means ± standard deviations (SD) (*n* = 6). Significance was determined by one-way analysis of variance (ANOVA) with the Duncan’s multiple-comparison test, and mean values without common letters indicate differences at *p* < 0.05.

The impacts of bovine α-LAH on the activation of IKK signaling pathway were determined. As shown in Figure 6A,B, HFD induced the increase of IKKα/β phosphorylation in epididymal adipose tissues. However, bovine α-LAH supplementation significantly reversed these changes. These results suggested that the supplementation of bovine α-LAH blocked the IKK signaling pathway activation in adipose tissues induced by HFD.

### 3.7. Bovine α-Lactalbumin Hydrolysates (α-LAH) Inhibited the Activation of MAPK Signaling Pathway in Epididymal Adipose Tissues of HFD-Fed C57BL/6J Mice

**Figure 7:**
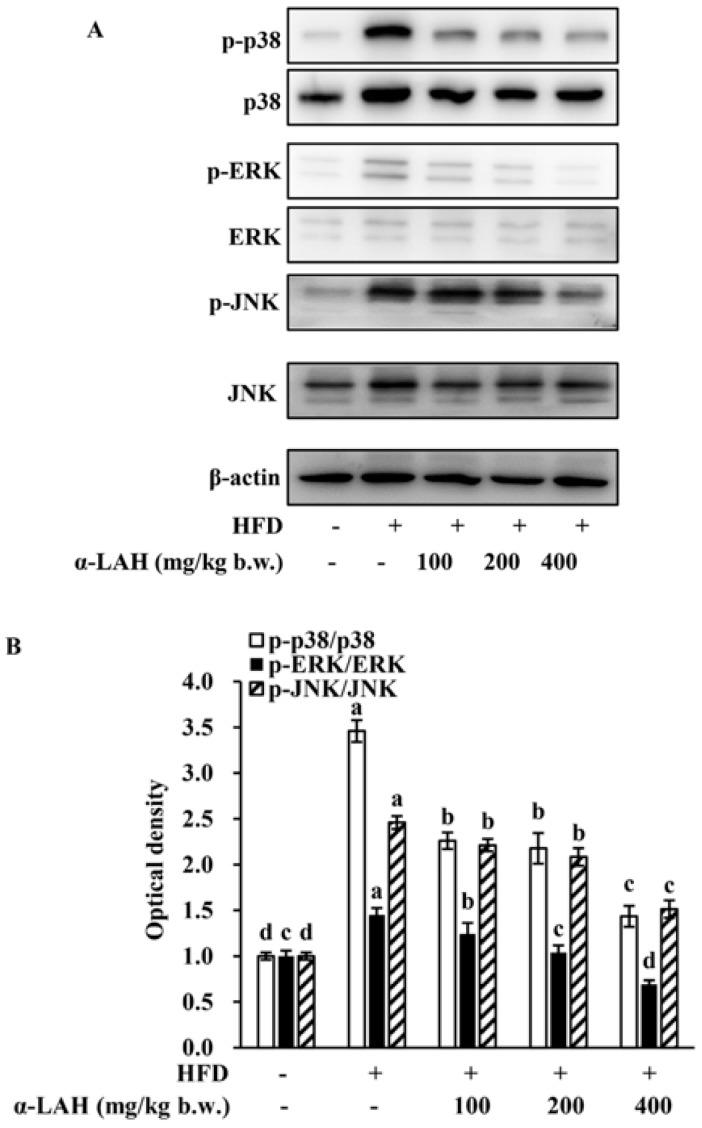
Effect of bovine α-lactalbumin hydrolysates (α-LAH) supplementation on the activation of mitogen activated protein kinase (MAPK) signaling pathway in epididymal adipose tissues of mice fed with high-fat diet. (**A**) A representative image of western blot analysis for p-p38, phospho-extracellular signal regulated kinases (p-ERK) and phospho-Jun N-terminal kinases (p-JNK) in epididymal adipose tissues; (**B**) fold change in relative densitometric levels of p-p38/ p38, p-ERK/ERK and p-JNK/JNK relative to the ND group. All data were presented as means ± standard deviations (SD) (*n* = 6). Significance was determined by one-way analysis of variance (ANOVA) with the Duncan’s multiple-comparison test, and mean values without common letters indicate differences at *p* < 0.05.

To elucidate potential protective mechanisms of bovine α-LAH against the high-fat diet induced insulin resistance and inflammation, the impacts of bovine α-LAH on the activation of MAPK signaling pathway in epididymal adipose tissues were determined. As shown in Figure 7, the HFD led to p38, ERK and JNK phosphorylation in epididymal adipose tissues. However, the supplementation of bovine α-LAH markedly decreased the levels of p-p38, p-ERK and p-JNK, thus blocking the MAPK signaling pathway (Figure 7A,B).

## 4. Discussion

When mice were fed with HFD for a period of time, it will develop obesity, hyperglycemia, hyperinsulinemia, and glucose intolerance, resulting in a state of insulin resistance [11]. In the present study, an insulin resistance model of C57BL/6J mice were established by feeding with HFD (60% kcal from fat) for eight weeks. Next, the mice were fed with HFD and bovine α-LAH for another 12 weeks to evaluate the protective effects of bovine α-LAH against HFD-induced insulin resistance. Our results showed that bovine α-LAH supplementation improved systemic and adipose insulin resistance, downregulated TNF-α, IL-6, and MCP-1 mRNA expression and suppressed IKKα/β and MAPK signaling pathways in adipose tissues of obese C57BL/6J mice induced by HFD.

Bovine protein and protein hydrolysates have been demonstrated to regulate glucose homeostasis induced by HFD. A hydrolysate prepared by bovine casein attenuated nod-like receptor protein 3 (NLRP3) activity and improved insulin signaling, resulting in reduced hyperglycemia and inflammation in mice fed with HFD [32]. Oral administration of bovine α-lactalbumin effectively improved glucose tolerance, enhanced high molecular weight form of adiponectin, and suppressed prostaglandin E2 levels in plasma in Goto–Kakizaki rats with type 2 diabetes [33]. Similarly, in our present study, bovine α-LAH significantly reversed the increase of body weight, fasting blood glucose, fasting serum insulin, HOMA-IR, OGTT AUC and ipITT AUC induced by HFD. These data suggested that bovine α-LAH administration ameliorated HFD induced systemic insulin resistance in mice. However, there was no significant difference in food intake among all the bovine α-LAH supplemented groups and HFD group, indicating that bovine α-LAH may lead to change in energy expenditure in mice fed with HFD. In previous studies, brown adipose tissue activity, physical activity, and gut microbiome are demonstrated to be related to energy metabolism in vivo, which will further induce change in body weight [34,35,36]. Thus, we speculate that bovine α-LAH might reverse HFD induced body weight gain through increasing brown adipose tissue activity, enhancing physical activity, or modulating gut microbiome, which awaits further investigation.

In the initiation and development of insulin resistance, adipose tissue has been considered to play an important role [37]. In the state of adipose insulin resistance, normal levels of insulin are unable to stimulate adipose glucose and lipid uptake and suppress triglyceride hydrolysis, due to inability of insulin to stimulate the down-stream IRS/PI3K/Akt pathway [38,39,40]. The insulin receptor (IR) activated by insulin leads to phosphorylation of insulin receptor substrate (IRS) on multiple tyrosine (Tyr) residues, followed by activation of PI3K and Akt. Activated Akt is capable of initiating glucose uptake, glycogen synthesis, lipid synthesis and protein synthesis in adipose tissue, muscle and liver [41,42,43]. However, phosphorylation of IRS-1 on serine (Ser) residues significantly reduces the function of IRS-1. The phosphorylation of IRS-1 on Ser307, which could uncouple IRS-1 from IR, negatively regulate tyrosine phosphorylation [13]. Moreover, phosphorylation of Ser612, which is located close to the tyrosine residues needed for PI3K binding, interferes IRS-1 stimulated PI3K activation [44]. In a previous study, corosolic acid improved insulin signaling in adipose tissues of HFD mice, evidenced by reduced IRS-1 serine phosphorylation (Ser307), increased IRS-1 tyrosine phosphorylation and Akt phosphorylation, leading to the improvement of glucose disposal [45]. Consistent with that, in the current study, bovine α-LAH reduced serine phosphorylation of IRS-1 (Ser307, Ser612) and enhanced serine phosphorylation of Akt (Ser473) in adipose tissue of HFD mice (Figure 4), indicating that bovine α-LAH improved HFD induced adipose tissue insulin resistance through IRS/PI3K/Akt pathway.

Inflammation may be an important contributor to HFD induced insulin resistance. In obesity, the hypertrophied adipocytes produce proinflammatory cytokines including MCP-1, TNF-α and IL-6 [46]. These cytokines cause recruitment of macrophages into adipose tissue, which will in turn enhance adipose tissue inflammation. These mechanisms cause a state of low-grade chronic inflammation, which has been proposed to be an important link between obesity and insulin resistance [47]. In a previous study, CCR2 inhibitor propagermanium significantly reduced the blood glucose, serum insulin, and HOMA-IR in HFD fed mice. Along with that, the increase of pro-inflammatory M1 macrophage markers (CD11c and MCP-1) expression in adipose tissue was also reversed [48]. Similarly, in the present study, bovine α-LAH not only exhibits protective effects against HFD induced insulin resistance, but also significantly downregulated inflammation-related gene (MCP-1, TNF-α and IL-6) expression in epididymal adipose tissues (Figure 5). These data suggested that the capacity of bovine α-LAH to improve insulin resistance may be related to their anti-inflammatory activities.

Inflammation and insulin resistance cross-talk through IKK and MAPK signaling pathways. A previous study demonstrates that insulin resistance in obese rodents could be ameliorated by inhibiting IKKβ activity or deleting IKKβ gene [14]. The mechanism by which IKKβ causes insulin resistance is that it could directly enhance IRS-1 phosphorylation on Ser307, a key inhibitory site of IRS/PI3K/Akt pathway [49]. Apart from IKKβ, MAPK signaling pathways are associated with insulin resistance and inflammation. The MAPK subfamily members, including ERK, JNK and p38 MAPK, could stimulate the production of pro-inflammatory cytokines, which will in turn contribute to the further activation of MAPK signaling pathways [42]. Among all of the MAPK subfamily members, JNK could promote the phosphorylation of IRS-1 on Ser307, whereas ERK could promote the phosphorylation of IRS-1 on Ser612 [13]. The serine phosphorylation decreases tyrosine phosphorylation of IRS-1, followed by negative modulation of the PI3K and Akt phosphorylation [41]. Given that bovine α-LAH reversed the increase of IRS-1 phosphorylation (Ser307 and Ser612) and inflammation-related gene (TNF-α, MCP-1 and IL-6) expression in epididymal adipose tissues induced by HFD (Figure 4 and Figure 5), we speculated that bovine α-LAH might ameliorate insulin resistance and inflammation through inhibiting IKK and MAPK signaling pathways. As expected, bovine α-LAH significantly suppressed phosphorylation of IKKα/β, JNK, ERK and p38 (Figure 6 and Figure 7), which provide compelling evidence that bovine α-LAH effectively interferes with the IKK and MAPK signaling pathways in adipose tissues, thereby contributing to its protective effects against insulin resistance and inflammation. However, the precise mechanism by which bovine α-LAH inhibits the IKK and MAPK signaling pathways, remains unknown and needs to be investigated in future studies.

## 5. Conclusions

In summary, our present study demonstrated that bovine α-LAH possesses protective effects against HFD induced adipose insulin resistance and inflammation in C57BL/6J mice. Bovine α-LAH also suppresses IRS-1 phosphorylation, enhances Akt phosphorylation, and inhibits the activation of IKK and MAPK signaling pathways in the adipose tissues of HFD-fed C57BL/6J mice, which may contribute to its protective effects against insulin resistance and inflammation. These findings suggest that bovine α-LAH may have the potential to be developed into functional foods against insulin resistance and inflammation associated with obesity.

## Figures and Tables

**Table 1:** Primers used for RT-qPCR.

Gene	Primer	Sequence (5′-3′)
TNF-α	Forward prime	CATCTTCTCAAAATTCGAGTGACAA
	Reverse prime	TGGGAGTAGACAAGGTACAACCC
IL-6	Forward prime	ATGGATGCTACCAAACTGGAT
	Reverse prime	TGAAGGACTCTCTGGCTTTGTCT
MCP-1	Forward prime	GCCCCACTCACCTGCTGCTACT
	Reverse prime	CCTGCTGCTGGTGATCCTCTTGT
GAPDH	Forward prime	TGGCAAAGTGGAGATTGTTGC
	Reverse prime	AAGATGGTGATGGGCTTCCCG

TNF-α, tumor necrosis factor alpha; IL-6, interleukin 6; MCP-1, monocyte chemoattractant protein 1; GAPDH, glyceraldehyde 3-phosphate dehydrogenase.
